# Percutaneous versus open posterior stabilization and decompression in AOSpine-type A3 thoracolumbar fractures with neurological deficit

**DOI:** 10.1186/s12891-023-06486-8

**Published:** 2023-05-15

**Authors:** Xin Song, Donglin Ren, Feng Zhang, Shuai Han, Desheng Wu, Jian Wang

**Affiliations:** 1 The Department of Orthopaedics, Pudong New District Peoples’ Hospital, Shanghai, China; 2 The Department of Spine Surgery, Shanghai East Hospital Affiliated to Tongji University, Shanghai, China

**Keywords:** Minimally invasive spine surgery, Decompression, Reduction, Neurological deficit, Percutaneous pedicle screw fixation (PPSF), Thoracolumbar fracture

## Abstract

**Background:**

This retrospective cohort study aimed to compare the clinical and radiological outcomes between two treatment strategies focusing on non-osteoporotic AOSpine-type A3 fractures of the thoracolumbar spine with neurological deficits at levels T11 to L2.

**Methods:**

In total, 67 patients between 18 and 60 years of age who were treated operatively with either of the two treatment strategies were included. One treatment strategy included open posterior stabilization and decompression, whereas the other was based on percutaneous posterior stabilization and decompression via a tubular retraction system. Demographic data, surgical variables, and further parameters were assessed. Patient-reported outcomes (PROs), including the Visual Analog Scale (VAS), the Oswestry Disability Index (ODI), and the American Spinal Injury Association (ASIA) impairment score, were measured to assess functional outcomes. The regional Cobb angle (CA), the anterior height ratio of the fractured vertebrae (AHRV), and the degree of canal encroachment (DCE) were assessed. The ASIA score was used to assess neurological function recovery. The follow-up period was at least 12 months.

**Results:**

Surgical time and postoperative hospital stay were significantly shorter in the minimally invasive surgery (MIS) group. Intraoperative blood loss was significantly less in the MIS group. Regarding radiological outcome, CA and AHRV at the time of follow-up did not show a significant difference. DCE at the time of follow-up was significantly improved in the MIS group. Lower VAS scores and better ODIs were observed in the MIS group at the 6-month follow-up, but similar outcomes were observed at the 12-month follow-up. The ASIA score was similar between both groups at the 12-month follow-up.

**Conclusions:**

Both treatment strategies are safe and effective; however, MIS could provide earlier pain relief and better functional outcomes compared with OS.

## Introduction

Thoracolumbar fracture (TLF) is the most common fracture of the spine [1], and approximately 20% of TLF cases are accompanied by neurological deficits [2]. The principal treatment modality for TLF with neurological deficits is surgery [2–6], as surgical intervention for TLF with neurological deficits not only provides spine stability and restores sagittal alignment, but most importantly also decompresses the neurological elements for the preservation of function.

Over the past three decades, transpedicular fixation has become the main strategy to stabilize the fractured segment and the adjacent normal segment. With the rapid development of minimally invasive instruments, percutaneous pedicle screw fixation (PPSF), introduced by Magerl in 1977, has been widely applied for TLF treatment because of advantages including decreased intraoperative blood loss, lower morbidity, less perioperative pain, accelerated ambulation, and faster return to work [7].

A variety of minimally invasive decompression instruments have been used for decompression of lumbar disorders, such as minimally invasive surgery–transforaminal lumbar interbody fusion (MIS-TLIF) performed in lumbar spinal stenosis using a tubular retraction system [8–10]. With the aid of illumination through a tubular retraction system, the operative area can be exposed clearly, and decompression can be performed securely.

To the best of our knowledge, there is no published study reporting simultaneous decompression and reduction of the intraspinal fracture fragments under direct vision through a tubular retraction system.

As a minimally invasive treatment method for degenerative lumbar diseases, a posterior midline approach using an intraspinal tubular retraction system can directly achieve spinal decompression using special tools similar to traditional surgical routes.

Based on these foundations, we report a novel technique for the treatment of A3 AO-type TLF with neurological deficits, which consists of PPSF, decompression of the spinal canal, and reduction of the intraspinal fractured fragment using a tubular retraction system via a posterior midline approach. This technique causes minimal procedure-associated trauma and can satisfactorily reduce the posterior vertebral wall fractured fragment under direct vision through a tubular retraction system in patients with A3 AO-type TLF and neurological deficits.

In the present study, we reviewed 67 cases of A3 AO-type TLF with neurological deficits treated with conventional open surgery (OS) or our novel MIS method and compared the clinical outcomes of the two procedures.

## Patients and methods

### Study design

A retrospective cohort study was conducted on 67 TLF patients who underwent either OS or MIS at a single academic institution between 2018 and 2020. All patients were followed up for at least 12 months. Informed consent was obtained from all patients. This study was approved by the institutional ethics committee of Shanghai Pudong New Area People’s Hospital (approval number: prylz2020-080) and conducted following the Declaration of Helsinki.

The inclusion criteria were as follows: (1) patients were aged 18–60 years; (2) patients were admitted within 24 h and received operation within 72 h after injury; (3) patients were diagnosed as having fresh single-level TLF (T11–L2, A3 type according to the AO classification [11]) by CT and MR imaging; (4) patients had a thoracolumbar injury classification and severity score (TLICS) of ≥ 4; and (5) patients had neurological deficits with spinal canal encroachment.

The exclusion criteria were as follows: (1) a former surgical history for vertebral fracture; (2) infections; (3) osteoporosis; (4) lamina fracture; (5) patients with preexisting neurological disorders; and (6) concurrent diseases including coagulation disorders, stroke, and tumors.

Finally, 67 patients were included. They were assigned to the following two groups: the OS group (*n* = 35) and the MIS group (*n* = 32).

### Data information

Demographic variables, including age, sex, and injured level, were collected. Surgical variables included operation duration, estimated blood loss (EBL), length of postoperative hospital stay, and complications.

Patient-reported outcomes (PROs), including the Visual Analog Scale (VAS), the Oswestry Disability Index (ODI), and the American Spinal Injury Association (ASIA) impairment scale [12], were collected pre- and postoperatively at 3, 6, 12, and 24 months.

Medical and surgical complications and the need for reoperation at the most recent follow-up were also gathered.

**Fig. 1:**
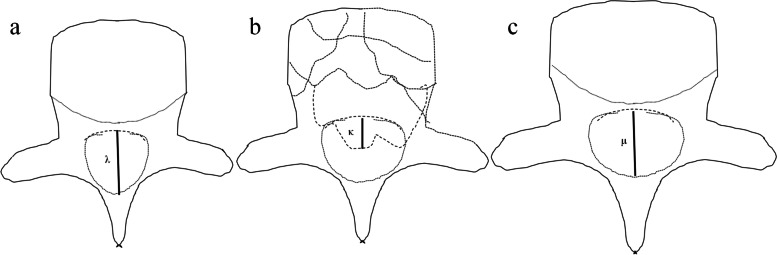
**a** λ = the anteroposterior diameter of the vertebral canal at the pedicle plane in the proximal adjacent segment of the fractured vertebra. **b** κ = the maximum anteroposterior diameter of the fracture fragments in the vertebral canal of the fractured vertebra. **c** μ = the anteroposterior diameter of the vertebral canal at the pedicle plane in the distal adjacent segment of the fractured vertebra. Degree of canal encroachment = κ/(λ + μ)/2

Radiological parameters including the regional Cobb angle (CA) and the anterior height ratio of the fractured vertebrae (AHRV) were measured by lumbar lateral radiographs preoperatively, on day 1 after surgery, at 3, 6, and 12 months, and on the most recent postoperative radiographs. The degree of canal encroachment (DCE) was measured on axial CT scan views preoperatively and at 3 and 12 months using the following equation: anteroposterior (AP) length of the spinal canal occupied by the fracture fragment divided by the mean AP spinal canal diameter of the above and below adjacent intact vertebrae (Fig. 1a–c).

**Fig. 2:**
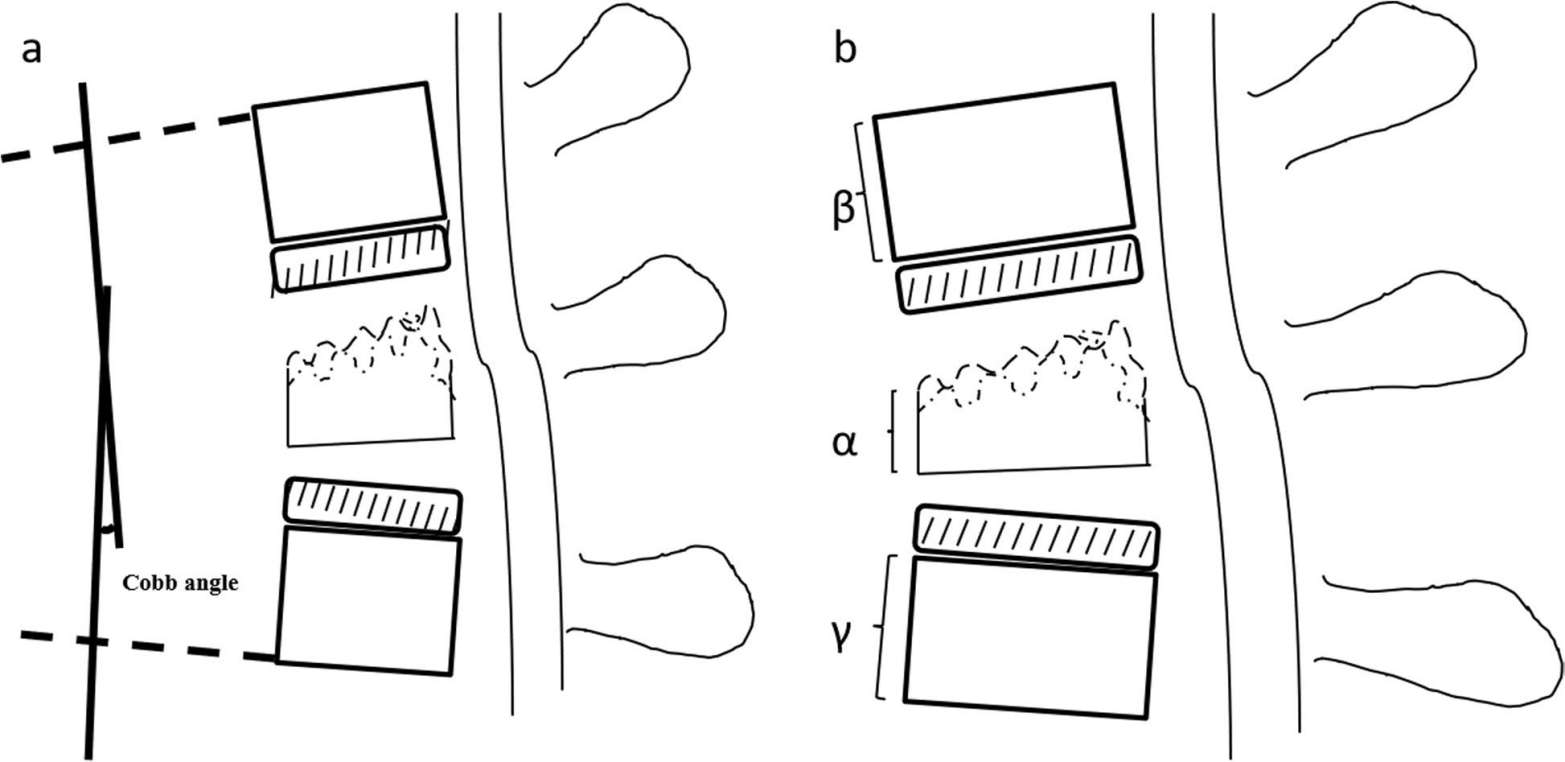
**a** Local kyphosis Cobb angle of fractured vertebra = angle of the vertical line of horizontal extension line of the upper endplate and the lower endplate on lateral X-ray. **b** Anterior height ratio of the fractured vertebrae = α/(β + γ)/2

The CA was measured from the line drawn on the upper endplate of the cranial adjacent vertebrae and the lower endplate of the caudal adjacent intact vertebrae by lateral plain radiography (Fig. 2a).

The anterior height ratio of the fractured vertebrae was calculated using the following equation: the vertebral body height of the fractured vertebra/the mean vertebral body height of the adjacent intact vertebrae by lateral plain radiography (Fig. 2b).

The ASIA grade was recorded preoperatively and at 3, 12, and 24 months.

### Surgical procedure

The operations in the MIS group were performed by one senior spine surgeon (Dr. Jiang Wang) and the operations in the OS group were performed by another senior spine surgeon (Dr. Xin Song). Under general anesthesia with endotracheal intubation, the patient was placed in a prone position on a radiolucent operation table. Manual reduction was conducted by the hyperextension method with two surgeons pulling the bilateral shoulders and legs to extend the entire spine. All surgeries were performed under neurophysiological monitoring.

### MIS group

#### Implantation of PPSF

1. From the AP view in the C-arm image intensifier, the entry points of PPSF were located at the 3 o’clock and 9 o’clock positions for the right-sided and left-sided pedicles of the fractured vertebra, as well as the adjacent vertebrae above and below. Then, a 15-mm longitudinal skin incision 10 mm lateral to the projection of the entry point was made and deepened into the underlying fascia.
2. The trocar was located through the incision at the entry point in the AP view and advanced into the vertebral body through the pedicle at a 15- to 30-degree medial angle. When the tip of the trocar approached the medial border of the pedicle on AP view, a lateral view was obtained. At this point, the trocar on lateral view should be at or slightly deeper than the posterior vertebral margin to avoid perforating the medial pedicle wall. After the trocar approached the middle of the vertebral body on lateral view, the guide wire was inserted and pushed into the vertebral body until 1 cm to the vertebral anterior edge.
3. A small dilation tube and protection tube were sequentially inserted over the guide wire. A self-tapping cannulated screw with proper diameter and length was inserted into the vertebra over the guide wire within the protection tube, and subsequently, the guide wire and protection tube were removed. Other pedicle screws were inserted in the same way under the monitor of the C-arm imaging intensifier.
4. The screw tail that penetrated the injured vertebrae was slightly higher than those placed on the adjacent segments of the injured vertebrae. Two polyaxial screws were placed in the injured vertebrae, and four monoaxial screws were used in the adjacent normal vertebrae.
5. The connection rod was prebended following the curvature of mild lordosis and inserted through the caudal incision to reach the cranial pedicle screw. Then, the screw nuts were locked sequentially to fix and reduce the injured vertebrae.

#### Decompression under a tubular retraction system

1. A 3–4-cm posterior midline skin incision was made according to the position of intracanal fracture fragments. The underlying spinous process was partly removed along with the adjacent supraspinous ligament and interspinous ligament.
2. The paravertebral muscles were bluntly dissected with sequential dilators, and then the tubular retraction system was implanted into the target site and fixed with a device mounted on the operation table. Under the illumination of the lighting system, the interlaminar space was identified clearly.
3. A proper laminectomy was performed with an ultrasonic osteotome (XDA860, Beijing Shui Mu Tian Peng Medical Devices Company, China) according to the level of intraspinal fracture fragments. After removing the ligamentum flavum, the bulged dura as an indicator for the location of the fracture fragment could be found (Fig. 3a), then reaching the front of the canal.

**Fig. 3:**
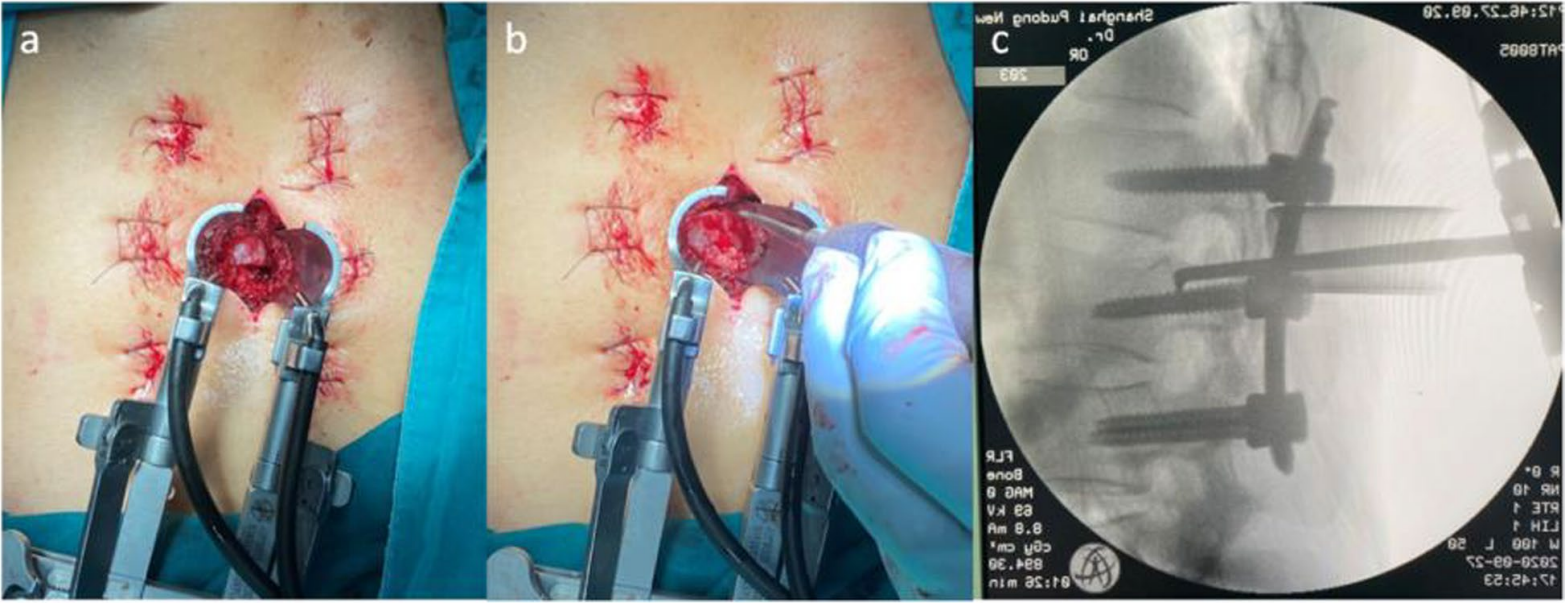
**a** Identification of the bulged dura after laminectomy using a tubular retraction system. **b** Placement or repositioning of the L-shaped retractor on the surface of the intraspinal fractured fragment through a tubular retraction system. **c** Satisfactory reduction of the vertebral body posterior edge by the L-shaped paddle retractor as verified by the intraoperative C-arm imaging intensifier
4. The dura and nerve root were carefully protected by gently using a brain cotton sheet and a nerve root retractor, respectively. A dura probe and a nerve root retractor were used to explore the posterior wall of the canal. After exploring along the posterior wall of the fractured vertebra to identify the position and the extent of the intraspinal fracture fragments, the possible adhesion between the fracture fragments and the ventral dura was carefully separated. Then an L-shaped tamp was inserted into the canal along the exploratory route. After slightly adjusting the L-shaped tamp to allow the angled tip to move onto the intraspinal fracture fragments and below the ventral dura, we gradually and repeatedly put pressure to reduce the major intraspinal fracture fragments and accomplish complete reduction. The tiny intraspinal fracture fragments were removed using forceps. Adequate reduction of the fracture fragments was confirmed by lateral view of the fluoroscopy (Fig. 3b–c). The surgical procedure is illustrated in Fig. 4a–b.

**Fig. 4:**
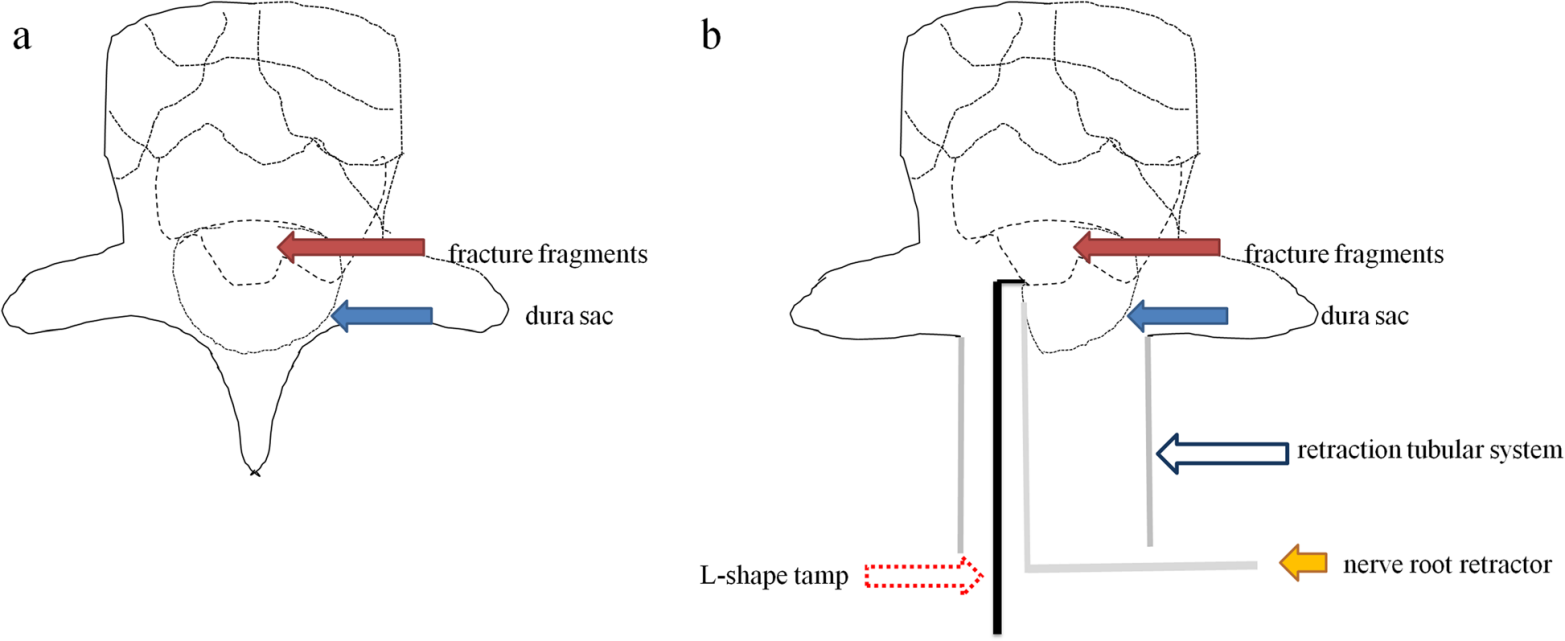
**a** Fracture fragments are indicated by a red solid arrow, and the dura sac is illustrated by a blue solid arrow. **b** After laminectomy, a retractor tubular system was implanted (blue hollow arrow); the dura sac was protected using a nerve root retractor (yellow solid arrow); an L-shaped tamp was applied for the reduction of major intraspinal fracture fragments
  During this procedure, neurophysiological monitoring should be performed to check for deterioration of neurological function. Sometimes dural tear happened resulting from the fracture; in these cases, we used a 5–0 suture to repair the dural sac.
5. After careful hemostasis, the wound was irrigated and closed in layers, with a drain placed around the decompression site.

OS group: OS was performed by a conventional posterior open procedure with a midline incision. This procedure was performed as described by Wu et al. [11].

#### Postoperative management

All patients received preventive antibiotic therapy within 24 h after surgery. The drainage tube was removed when the drainage blood volume was less than 30 ml within a day. All patients were allowed to leave their beds and supported by a hard brace until 7 days postoperation. After discharge, patients were transferred to a rehab center for further therapy. The hard brace was worn for 12 weeks. All patients were given oral methycobal for 3 months. All patients were routinely followed up.

### Statistical analysis

Statistical analysis was performed using SPSS software (Version 19.0). Continuous variables are shown as mean ± standard deviation (SD). To compare the operation parameters between two groups, an independent t-test was used. A paired t-test was used to compare preoperation and postoperation parameters in each group. The chi-square test was used to compare categorical variables. A two-sided *P*-value of < 0.05 was considered to indicate statistical significance.

## Results

### Perioperative information

The operation was accomplished in all 67 patients. PPSF was precisely performed without breakage of the medial pedicle wall according to postoperative CT scans. The average follow-up period was 24.7 months (range, 18–35 months) and 20.2 months (range, 12–30 months) in the OS and MIS groups, respectively. No significant differences in age, gender, and injured level were observed between the two groups (*P* > 0.05) (Table 1). Operation duration in the OS and MIS groups was 172.80 ± 33.14 min and 125.56 ± 23.53 min, EBL was 238.51 ± 31.36 ml and 118.41 ± 19.31 ml, and length of postoperative hospital stay was 15.26 ± 3.68 days and 12.51 ± 2.61 days, respectively (Table 1).

**Table 1:** Demographic data

	MIS group	OS group	*P*-value
Number	32	35	
Age	41.32 ± 8.18	39.86 ± 7.31	0.442
Sex (male/female)	20/12	24/11	0.601
Injured level			
T10	2	1	
T11	5	4	
T12	14	15	
L1	8	10	
L2	3	5	
TLICS score			
5	15	17	
6	5	4	
7	7	9	
8	5	5	
Operation duration (min)	125.56 ± 23.53	172.80 ± 33.14	< 0.001*
Estimated blood loss (ml)	118.41 ± 19.31	238.51 ± 31.36	< 0.001*
Postoperative hospital stay (days)	12.51 ± 2.61	15.26 ± 3.68	< 0.001*

^*^ *P* < 0.05

Operation duration and length of postoperative hospital stay were significantly shorter in the MIS group than in the OS group (*P* < 0.05). EBL was less in the MIS group than in the OS group (*P* < 0.05) (Table 1).

### Radiological outcomes

**Fig. 5:**
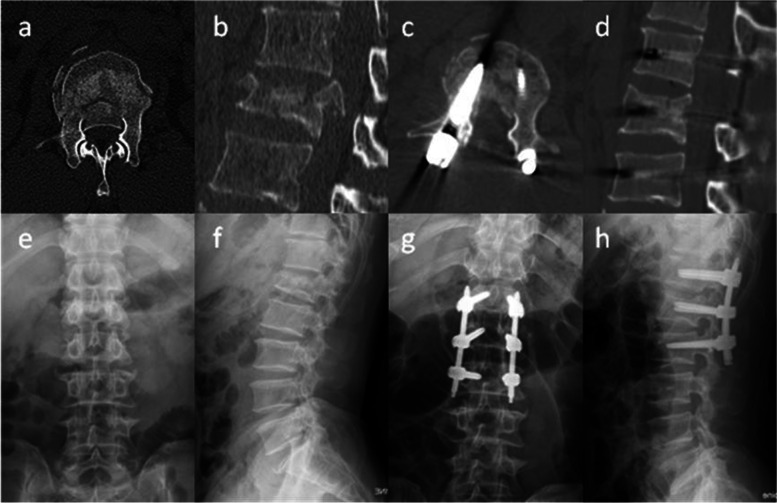
**a**–**b** Severe spinal canal encroachment as demonstrated by preoperative axial CT scan and sagittal reconstruction imaging. **c**–**d** Satisfactory improvement of spinal canal encroachment as demonstrated by postoperative axial CT scans and sagittal reconstruction imaging. **e**–**f** Lumbar AP and lateral X-ray preoperatively. (**g–h**) Lumbar AP and lateral X-ray postoperatively

CA, AHRV, and DCE were significantly improved after surgery in both groups, and these were well maintained until the last follow-up (*P* < 0.05). There were no significant differences in CA and AHRV between the two groups during follow-up (*P* = 0.356 and *p* = 0.249, respectively); however, postoperative DCE was significantly better in the MIS group than in the OS group (*P* < 0.05) (Fig. 5a–h; Table 2).

**Table 2:** CA, AHRV, and DCE

	Time point	MIS group	OS group	*P*-value
CA (°)	Preoperative	21.56 ± 6.39	22.89 ± 5.16	0.350
	Postoperative 3 months	6.68 ± 1.68	7.33 ± 0.94	0.059
	Last follow-up	8.51 ± 1.89	8.90 ± 1.60	0.356
	Correction loss	1.83 ± 0.56	1.58 ± 1.16	0.254
*P*-value		< 0.001*	< 0.001*	
AHRV (%)	Preoperative	73.74 ± 6.45	74.62 ± 7.40	0.629
	Postoperative 3 months	89.17 ± 6.35	87.74 ± 2.92	0.251
	Last follow-up	87.54 ± 6.41	86.10 ± 2.87	0.249
	Correction loss	1.63 ± 0.71	1.64 ± 1.12	0.951
*P*-value		< 0.001*	< 0.001*	
DCE (%)	Preoperative	46.06 ± 8.90	44.50 ± 6.59	0.414
	Postoperative 3 months	12.20 ± 2.55	15.20 ± 1.81	< 0.001*
	Last follow-up	12.51 ± 2.54	15.72 ± 1.74	< 0.001*
	Correction loss	0.31 ± 0.22	0.52 ± 0.44	0.014*
*P*-value		< 0.001*	< 0.001*	

*CA* Cobb angle, *AHRV* Anterior height ratio of the fractured vertebrae, *DCE* Degree of canal encroachment

^*^ *P* < 0.05

### Clinical outcomes

With respect to neurologic recovery in terms of the ASIA grade, in the MIS group, six patients achieved one-grade recovery from C to D, 17 patients achieved one-grade recovery from D to E, five patients achieved two-grade recovery from C to E, and four patients remained at the same level at grade C or D at the last follow-up. In the OS group, nine patients achieved one-grade recovery from C to D, 15 patients achieved one-grade recovery from D to E, five patients achieved two-grade recovery from C to E, and six patients remained at the same level at grade C or D at the last follow-up (Table 3).

**Table 3:** Neurologic outcomes according to ASIA classification

	ASIA grade (last follow-up)			
		C	D	E
MIS group				
ASIA grade (preoperative)	C	1	6	5
	D		3	17
OS group				
ASIA grade (preoperative)	C	1	9	5
	D		5	15

There was no statistically significant difference in preoperative ASIA scores between the OS and MIS groups (*P* = 0.655). Follow-up also showed no statistically significant difference in ASIA scores between the two groups (*P* = 0.592).

Both VAS scores for back pain and ODI scores were not significantly different between the OS and MIS groups preoperatively (*P* > 0.05). During follow-up, these were significantly improved in both groups (*P* < 0.05). At 3 and 6 months, VAS scores were significantly better in the MIS group than in the OS group (*P* < 0.05); however, there was no significant difference in VAS scores at the last follow-up between both groups (*P* = 0.301). Similar results were observed for the ODI score (Table 4).

**Table 4:** VAS and ODI

	Time point	MIS group	OS group	*P*-value
VAS	Preoperative	7.94 ± 0.72	7.94 ± 0.62	0.836
	Postoperative 3 months	2.88 ± 0.66	3.66 ± 0.54	< 0.001*
	Postoperative 6 months	1.94 ± 0.50	2.80 ± 0.47	< 0.001*
	Last follow-up	1.53 ± 0.51	1.66 ± 0.48	0.301
*P*-value		< 0.001*	< 0.001*	
ODI	Preoperative	68.72 ± 2.37	69.89 ± 3.35	0.103
	Postoperative 3 months	31.75 ± 1.69	29.03 ± 2.88	< 0.001*
	Postoperative 6 months	20.94 ± 2.81	18.77 ± 2.50	0.001*
	Last follow-up	15.81 ± 2.31	16.31 ± 1.92	0.335
*P*-value		< 0.001*	< 0.001*	

*VAS* Visual Analog Scale, *ODI* Oswestry Disability Index, *CA* Cobb angle, *AHRV* Anterior height ratio of the fractured vertebrae, *DCE* Degree of canal encroachment

^*^ *P* < 0.05

### Intraoperative and postoperative complications

In the OS group, two cases of dural tear resulting from the fracture occurred; in these cases, we used a 5–0 suture to repair the dural sac. Moreover, one screw-rod failure and one postoperative infection were reported in the OS group. Screw failure was treated conservatively due to the absence of clinical symptoms, and postoperative infection was treated by debridement operation. All four patients with complications showed favorable outcomes at the most recent follow-up. In the MIS group, one case suffered from nerve root trapping within the lamina at the decompression step. We released the trapped nerve root under the tubular system without reducing neurological function. One case of screw loosening was reported in the MIS group and managed conservatively. The patient remained clinically silent at the most recent follow-up. There were no cases of deterioration of neurological function in both groups.

## Discussion

Current surgical options for TLF with neurological deficits include posterior pedicle screw fixation combined with posterior laminectomy, anterior fixation combined with anterior direct decompression, and anterior decompression and fixation combined with posterior fixation [3, 5, 6, 12–14]. Although fixation and decompression via the anterior approach can achieve anterior column support and direct decompression [15], it is less used because posterior transpedicular approaches to the anterior column are safer and easier [16].

As a minimally invasive technique, PPSF is widely applied in posterior fixation of spinal fractures with similar fixation effects to traditional open posterior pedicle screw fixation but with less trauma and perioperative blood loss [17].

In the current study, operation duration (average, 125.56 min) and postoperative hospital stay (average, 12.51 days) were much shorter and EBL (average, 118.41 ml) was much less in the MIS group than in the OS group (average: 172.80 min, 15.26 days, and 238.51 ml, respectively). These perioperative parameters were in accord with former studies and associated with faster postoperative recovery.

In patients with A3-type spinal fractures, an individual posterior approach may achieve satisfactory clinical effects without further decompression and reduction of spinal fractures [18]. However, it is standard of care to decompress the spinal canal in patients with neurological injury, as that is the only way to restore function.

The invasiveness and feasibility of decompression methods must be taken into account, but whether the intraspinal fractured fragment should be reduced or not remains unclear. To the best of our knowledge, the reduction of intraspinal fracture fragments via a posterior approach was popularized by indirect decompression methods including postural reduction or instrumental reduction based on the integrity of the posterior longitudinal ligament. Nevertheless, unsuccessful reduction of intraspinal fracture fragments due to disruption of the posterior longitudinal ligament is not uncommon, especially in patients with neurological deficits.

Tang et al. [19] analyzed the independent risk factors of neurological deficits after thoracolumbar burst fracture and found that the degree of spinal canal encroachment and the anterior vertebral compression ratio were independent variables associated with neurological deficits.

In agreement with the above risk factors, intraspinal remnant fracture fragments are common in postoperative CT scans in patients without satisfactory recovery of neurologic function according to our clinical observations. Therefore, proper reduction of intraspinal fracture fragments should facilitate the recovery of neurologic function. Furthermore, rebuilding the anatomic continuity of the vertebral body posterior edge would impact the restoration and maintenance of the height of the fracture vertebral body.

In the first step of our operative procedures, PPSF acquired satisfactory radiological parameters similar to the OS group and previous studies [12, 15, 17, 18]. Based on the postural and instrumental reduction, the anterior height ratio of the fracture segment improved from preoperative (73.74 ± 6.45)% to postoperative (89.17 ± 6.35)% and remained at (87.54 ± 6.41)% at the last follow-up; sagittal alignment improved from preoperative (21.56 ± 6.39)° to postoperative (6.68 ± 1.68)° and remained at (8.51 ± 1.89)° at the last follow-up. Although these parameters were similar to those in the OS group, a better trend was observed in the MIS group.

In the second step, accurate laminectomy was successfully performed through a tubular retraction system in accord with the corresponding location of the intraspinal fracture fragment. Through the operative window made by laminectomy, the L-shaped retractor was cautiously placed into the front of the spinal canal, and the intraspinal fractured fragment was repositioned properly under direct vision. Postoperative CT scans demonstrated that DCE substantially improved from preoperative (46.06 ± 8.90)% to postoperative (12.20 ± 2.55)% and remained at (12.51 ± 2.54)% at the last follow-up. At postoperative follow-up, DCE was significantly better in the MIS group than in the OS group. The following reasons may contribute it. First of all, in the MIS surgery group, we will precisely locate the intravertebral fracture mass on preoperative CT, position the tunnel at the appropriate location intraoperatively, and reset the intravertebral fracture mass under direct vision after a small laminectomy. It may be due to the fact that more posterior bony structures of the vertebral body are preserved in the MIS surgery group, which helps to maintain the repositioning of the intracanal fracture mass. In addition, because the MIS surgical group has a smaller operative field than the open surgical group, the surgeon will use the intraoperative O-arm to assist in adjusting the position of the access for decompression in some cases where the degree of intraoperative intravertebral canal decompression is uncertain. As a consequence of these, our novel technique achieved a larger spinal canal volume according to the DCE in the MIS group but a similar status of neurological function recovery compared with the OS group. This may be caused by the relatively short follow-up; a longer follow-up period should be employed to further study neurological function recovery.

Results of clinical outcome showed superior improvement in terms of VAS and ODI scores in the MIS group compared with the OS group at 3 and 6 months postoperatively. However, at the last follow-up, there were no significant differences in VAS and ODI scores between the two groups. Therefore, early recovery of back pain and function was associated with the application of MIS techniques, and the extent of paraspinal muscle dissection could have an important impact on early clinical outcomes.

Dural tear is not unusual due to the intraspinal fracture fragment in patients with TLF, which was observed in two cases in the OS group in this study. It was solved using a 5–0 suture and recovered without clinical symptoms. One case with nerve root trapped within the lamina was found at the decompression step in the MIS group. Under neurophysiological monitoring, we released the nerve root without deterioration of neurological function.

Usually, patients with single-segment spinal fractures could achieve satisfactory clinical outcomes with fixation at only one level above and one level below without anterior column support. However, instrumentation failure was observed in one case in the OS group and one case in the MIS group. Earlier loading or osteoporosis may contribute to this.

In the OS group, screw breakage was observed in one patient at the 3-month follow-up. One screw loosening in the MIS group was observed below the fractured level at the 6-month follow-up. These two patients were closely followed up with radiological and clinical examinations. At the most recent follow-up, the two patients’ radiological outcomes with regional kyphosis and clinical outcomes were satisfactory, and no further surgical intervention was required. Based on careful evaluation, screw failures in these two patients could be caused by early physical activities.

Studies reporting simultaneous decompression and reduction of the intraspinal fractured fragment with MIS are scarce. Huang et al. [20] performed a two-stage operation for a case of A3-type L1 burst fracture. Following the principles of MIS, transforaminal endoscopic spinal canal decompression was applied for the case in the second phase with satisfactory recovery of neurologic function. However, second-phase transforaminal endoscopic spinal canal decompression may be performed before or after the optimal period for spinal cord decompression. In addition, it is difficult to implement hemostasis around the decompression site. Moreover, postoperative hematoma in the region of the thoracolumbar spinal canal may have disastrous consequences. For these reasons, larger-sample studies are necessary to verify the effects of these techniques.

Our novel decompression and reduction method can be performed for multiple spinal fracture types and multi-site decompression and reduction with the following advantages. First, the tubular retraction system can be placed anywhere, thus ensuring smooth and accurate decompression in line with the location of spinal canal encroachment. Second, the laminectomy size can be adjusted appropriately using a tubular retraction system according to the operational area for reduction. Third, hemostasis around the operational area can be easily achieved under auxiliary illumination using a tubular retraction system. Finally, with careful hemostasis and proper protection of the dura, the L-shaped retractor can be laid on the surface of the fractured fragment or repositioned securely under direct vision.

There are some limitations in our study. First, the study was performed in a single center with a relatively small sample size and a relatively short follow-up duration. In addition, follow-up observations after the removal of the hardware are lacking. Therefore, our novel surgical method needs to be verified by large-sample studies with longer follow-up periods.

## Conclusions

OS and MIS are safe and effective methods for the treatment of A3 AO-type TLF with neurological deficits. Although both groups showed favorable clinical and radiological outcomes at the final follow-up, MIS was less invasive, provided earlier pain relief, and resulted in a larger volume of the spinal canal compared to OS. This study indicates that ongoing use of PPSF combined with decompression and reduction using a tubular system is recommended for the treatment of A3 AO-type TLF with neurological deficits.

## Data Availability

The datasets used and/or analysed during the current study are available from the corresponding author on reasonable request.
